# Tourism and Urban Development: The case of a coastal village in a small island state

**DOI:** 10.12688/openreseurope.17641.1

**Published:** 2024-08-15

**Authors:** Karl Agius, Michael Briguglio

**Affiliations:** 1 University of Malta, Msida, Malta

**Keywords:** Maltese Archipelago; Sustainable Tourism; Blue Economy; Touristification; Real Estate; Maritime Sociology; Sustainable Development Goals; Coastal Tourism

## Abstract

This paper discusses how Marsascala, a coastal town in Malta, has been urbanized over the years, with particular attention to the development of the tourist industry. Young’s (1983) “general model of the process of ‘touristization’ and landscape change” is engaged with to study the impact of tourism development on the locality and local community. Different research methods were used, including qualitative interviews via thematic analysis, fieldwork, orthophoto maps, and analysis of secondary data. Findings show that tourism plays a major role in the local economy. Overdevelopment and population increase (fuelled by tourism and influx of foreign workers) are major challenges in the locality. The former fishing village has as a result reached stage six (intensive tourism consolidation) of Young's model. While more services are available to locals and visitors, the quality of life in the locality is deteriorating. This has raised questions about the need to redevelop the Jerma Hotel which will partly be a real estate project. The authors propose a seventh stage to Young’s model - ‘real estatation’ whereby more areas are taken over by real estate projects including for short-term rentals. This paper is linked to the EU Cost Action CA221222 Rethinking the Blue Economy: Socio-Ecological Impacts and Opportunities (RethinkBlue), in particular in relation to the themes covered by Working Group 3 - Port cities & coastal communities.

## Introduction

Tourism is a vital economic sector in the Mediterranean basin. In particular, coastal tourism is a major sector in the Blue Economy. In its Blue Economy Report for 2023, the European Commission states that the latter is linked to many other economic activities, thus having effects on employment, income, and well-being. From a macroeconomic perspective, the Blue Economy in general is considered to be a more significant contributor to national Gross Value Added (GVA) and employment in insular Member States or those with archipelagos, such as Malta (p.9). In its report, the European Commission notes that coastal and maritime tourism is the largest and fastest-growing sector of the EU Blue Economy, attracting many visitors to EU coastal areas.

At the same time, the Blue Economy paradigm is subject to critical engagement, for example in relation to its impacts and opportunities. A practical example of this is the EU COST Action CA221222 Rethinking the Blue Economy: Socio-Ecological Impacts and Opportunities (RethinkBlue), As per its Memorandum of Understanding ( COST, 2023), this COST Action centres around the Blue Economy and related policies affecting European societies. Its purpose is to ‘rethink the Blue Economy, in two ways. First, by assessing its impact on coastal societies, and second, by exploring opportunities deriving from innovations and potential synergies between established and emergent marine activities.’ (p.3)

One of the areas of focus of RethinkBlue is ‘Port Cities & Coastal Communities’ which is summed up as follows in the Memorandum of Understanding (p.9):

**Table:** 

WG3 "Port cities & coastal communities"	Port cities and coastal communities as complex organisations that are deeply connected with local spaces, culture, economy, and environment	• Tourism and recreational use of ports and coastal areas • Coastal demography and new inhabitants, e.g. labour migrants, maritime lifestyle migrants etc. • Conflicts between different users • Identity, maritime heritage and history: different uses of the past

In this paper, coastal tourism is situated accordingly. Here one needs to mention that apart from impacts such as economic growth, this sector also faces high seasonality ( Agius & Briguglio, 2021). This is not to mention that coastal zones also face the threat of sea level rise and storms due to climate change (p.37) ( European Commission, 2023). This industry's extensive and uncontrolled growth may negatively impact the fragile natural coastline ( Mejjad *et al.*, 2022). Several small settlements along the Mediterranean coast have experienced extensive urbanisation as a result of incentives that encourage tourism development. Construction of hotels as well as other housing projects have increased drastically. The dense construction of multi-storey buildings along the shoreline resulted in loss of green spaces, overpopulation and overstretching of local infrastructure ( Burak *et al.*, 2004).

The aim of this paper, which was presented in the first conference of the RethinkBlue Cost Action (2024) is to analyse how Marsascala, a coastal town in Malta, has been urbanized over the years, with particular attention to the development of the tourism industry. In this regard, Bruce Young’s (1983) “general model of the process of ‘touristization’ and landscape change” is engaged with, together with relevant theories and studies of tourism and urban development to understand which stage of tourism development Marsacala has reached, how has tourism development altered the locality, what are the implications on the local community and what are the next changes that the locality can undergo.

## Literature review

### Tourism and urban development

This section engages with social-scientific literature on the interaction between tourism and urban development. The concept of ‘touristification’ is often used to understand such social processes. It is often interpreted as the synthesis of ‘tourism’ and ‘gentrification.’ Ojeda and Kieffer (2020) appeal for an evidence-based phenomenological operationalisation of the touristification term, referring to a concrete actual geographical space, rather than an ideologically loaded a priori notion. Touristification here is seen as an empty signifier, which can be filled with the particular characteristics of the touristic territory in question. Some empirical examples will be discussed below. In turn they will inform this study, in conjunction with Young’s (1983) theoretical concept of touristization, which will also be referred to.

Some authors focus on the impacts of top-down initiatives in urban planning and tourism. For example, Andrade and Costa (2020) refer to the importance of spatial planning and its respective impacts on touristic development. Leccis (2023), discusses the interrelationship between urban planning and tourism development in Cagliari, Italy, and focuses on the negative impacts of overtourism, such as the transformation of entire neighbourhoods for touristic purposes. In turn, residents in Cagliari (and elsewhere) have organised protests against such development.

On the other hand, Adhinata and Sawitri (2022) consider how government policy and capitalist development provide significant opportunities in a new socio-economic climate which co-opts the local community to participate in the touristification process, for example through the development of different types of accommodation for such purposes. Thus, structure and agency are interrelated in their analysis of the changing of the use of spaces, from fishing and farming to tourism in Canggu, a village in Bali, Indonesia. On similar lines, Woo *et al.* (2022) find mixed impacts on residents’ quality of life in Buckchon Hanok Village, Seoul, South Korea.

Another approach, hereby exemplified by Freytag and Bauder (2018), highlights bottom-up processes, where tourists and tourism play instrumental roles in urban development. Their research, which is based in Paris, shows how the social proximity of tourists and residents influence each other. Tourist spaces are created, and policies such as the development of Airbnb extends such areas and related sites. Urban development, gentrification, and mobility interact with planning and are thus mutually constitutive.

Jover and Diaz-Parra (2020) emphasize the dialectical relationship between touristification and gentrification, in “that transnational gentrification and touristification are new urban strategies and practices to revalorise real estate and appropriate urban surplus in unique urban areas” (p.3044). In turn, they relate touristification to an increase in tourist activity that generally implies the loss of residents and authenticity, to accommodate the needs of tourists, who are temporary. For example, in their study based in Seville, Spain, Jover and Diaz-Parra find an increase of establishments such as cafés and nightclubs. On the other hand, they also observe gentrification, which they define as the replacement of low-income populations with new higher-income residents, including lifestyle migrants, in higher-priced residential property, which they also observe in their respective study.

Of particular interest to this study is Young’s (1983) model to showcase the transition that a locality’s landscape can go through with tourism development. In his study, he used the same locality under investigation in this article as his case-study. He examines how tourism transforms the landscape and culture of villages: he introduces the terms ‘touristization’ and ‘villscape’ to describe these changes. Young also proposes a general model of touristization and villagescape change, based on the examples of former fishing and farming villages.

The model shows how a village changes as tourism becomes its main activity, through different stages. Tourism starts as an extra activity (Stage 3) and eventually dominates (Stage 6) the village’s physical, economic, and cultural aspects. The model also shows how as of Stage 4, real estate starts to play a prominent role with the development of apartments which increases sharply by an increase in permanent residents (Stage 5).

It is also interesting to note that Milano *et al.* (2023) emphasize that tourism is not the only cause of structural change in places which are often deemed as being touristified. They refer to the impacts of temporary residents, digital nomads, international students, short-stay expats, creative workers, and others, often resulting in an increase in the price of housing and a loss of sense of belonging in respective neighbourhoods. In this regard, whilst important for policy makers, the evolutionary relationship between tourism and urbanisation is still undefined and requires further attention ( Kabil *et al.*, 2022).

### Tourism in Malta

Given that this study focuses on a locality in Malta, it is important to give a brief outline of the development of tourism on this small archipelago.

Since the country’s independence in 1964, successive governments have invested heavily in tourist infrastructure. The tourism product has also been enhanced to compete in a challenging market. Tourism numbers and expenditure have increased steadily, throughout the years, with the introduction of low-cost carriers bringing even higher tourist arrivals. Eventually, by the end of the century, concerns about the sustainability of the tourism sector were growing, for example in terms of overdevelopment, congestion, and environmental degradation ( Cassar, 2016).

Tourism was thus having both positive and negative effects on host communities. For example, it has been a key economic sector in the Maltese islands reaching 3 million tourists ( The Malta Independent (1)). In the meantime, the percentage of Maltese workers in the tourism sector decreased from 82% in 2009 to 40.6% in 2019. The sector thus had to rely on foreign workers due to the growth of the tourism sector and the limited local workforce. As a result, the Maltese islands experienced a construction boom in short-let private accommodation to respond to the demand in accommodation and the sector’s dependence on expats who require accommodation ( MHRA, 2022).

In the meantime, whilst the concept of sustainable tourism was mentioned in policy documents of different governments, “in practice however, sustainability was often mostly offered lip service only and the success of the industry was generally measured in terms of tourist numbers by the tourism authorities. The dependence of mass tourism continued unabated, and very little, if at all, was done to reverse this trend.” ( Briguglio & Avellino, 2021, p.134–5).

Besides, Malta did not manage to reach EU and national targets related to alternative energy ( Agius, 2022). Thus, the Maltese Islands have experienced large-scale building activity regardless of the environmental impact and the reduction of the basic attractive qualities of the island to future visitors ( Baldacchino, 2012).

The phenomenon of lack of sustainability was articulated through a perception survey related to people’s attitudes towards tourism on the islands, as well as whether Malta is experiencing ‘overtourism’ ( Briguglio & Avellino, 2021). Whilst the respondents highlighted the economic, socio-cultural, and infrastructural benefits of tourism, as well as the honour and pride of hosting tourists, they also expressed concerns about the environmental, social and economic costs of tourism, such as pollution, congestion, loss of identity and price inflation (p.137).

## Materials and methods

### Area of study

**Figure 1.**
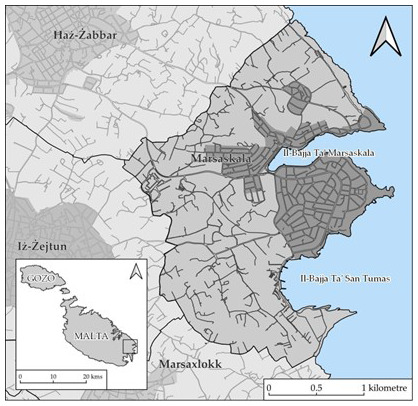
Map showing the locality of Marsascala. Map prepared for the authors by Andrea Pace.

This study focuses on Marsascala (see Figure 1), a main seaside town situated in Malta’s South-East situated between two valleys not far from the towns of Żabbar and Żejtun ( Malta Tourism Authority (1)).

The locality has witnessed a significant increase in its population over the years (see Table 1). According to Malta’s national census, the locality had a population of 888 in 1957, which grew to 1,936 in 1985, before increasing to 4,770 in 1995, 9,346 in 2005, 11,059 in 2011, and 16,804 in 2021. The current population consists of 12,157 Maltese and 4,647 foreigners. Between 1995 and 2021, the population density per square kilometre increased from 2,239, to 3,126. Comparatively, Malta’s population stood at 519,562, experiencing a massive increase of 25% in a decade, “the highest intercensal change ever recorded to date.” - and the population density was of 1,648.6 per square kilometre in the same year ( National Statistics Office – Malta (1)). The increase in residents in Malta, which has one of the lowest fertility rates in the European Union, was mainly due to foreign workers. In fact, more than one in five residents are foreign, with 115,449 non-Maltese persons residing in Malta. In the South-East district, where Marsascala is situated, the top five of non-Maltese nationalities are Italian (2,007), British (916), Indian (518), Filipino (510), and Serbian (494) ( National Statistics Office – Malta (3)) (see Table 2).

**Table 1.** Population of Marsascala and Malta according to Malta National Census.

Year	Marsascala	Malta
1921 1931 1948 1957 1967 1985 1995 2005 2011 2021	- - - 888 876 1,936 4,770 9,346 11,059 16,804	212,258 241,621 305,991 319,620 314,216 345,418 378,132 404,962 417,432 519,562

Source: NSO (1)

**Table 2.** Non-Maltese population.

District	Italian	British	Indian	Filippino	Other nationalities	Other EU Member States
**Malta** **South Eastern**	13,838 2,007	10,614 916	7,764 518	7,571 510	20,967 1,957	22,443 1,595

Source: NSO (3)

Going back to the mid-twentieth century, Marsascala was primarily a fishing village, and it has also served as a summer resort ( Seychell, 2010). Eventually, the locality was earmarked for touristic development - where it hosted a major four-star hotel - The Jerma Palace - between 1982 and 2007 ( Hopkins, 2014). This explains why as of the 1980s and 1990s the locality became a destination for both locals and tourists ( Malta Tourism Authority (1)).

Previous studies had shown that although a number of international tourists visited or resided temporarily in Marsascala, it was mainly domestic tourism which was responsible for the population increase felt mostly during the summer months ( Brincat, 2002). In the 2000s, the locality started to witness a decline in tourism, and this was attributed to a number of factors including the treatment of waste on the outskirts of Marsascala ( Azzopardi, 2007), which was itself mired in political controversy ( Aquilina, 2011).

Due to its restaurants, hotels/guest houses (see Table 3), bars and clubs, the locality has nowadays developed into a touristic hub and entertainment destination. The locality houses circa 30,000 people in the peak summer months and according to the Malta Tourism Authority’s (MTA) Traveller Survey, an estimated 270,000 tourists visited Marsascala in 2019 ( Malta Tourism Authority (1)).

**Table 3.** Licensed accommodation in Marsascala throughout 2023.

Type of accommodation	Licensed applications	Number of beds
Hotels Furnished premises Host families Total	2 39 7 48	104 163 20 287

Source: Malta Tourism Authority (2)

During the past decades, the locality was subject to much land and property development ( Times of Malta (1)), and in some cases it was the locality with the highest number of new dwellings approved by the Planning Authority ( National Statistics Office – Malta (2). Another visual example of social and economic changes in the locality is that there are many more pleasure craft anchored in its sheltered bay than fishing boats ( Baldacchino, 2012).

Other recent developments include the closing of the same waste treatment plant, the development of a family park, and the announcement that the former Jerma Palace Hotel in Marsascala would be redeveloped into a complex of 130 apartments and a 500-room hotel ( Times of Malta (3)). Marsascala has also been subject to environmental contention, for example on the respective proposed developments of a new University Campus, a water polo pitch and a yacht marina. These projects, as well as earlier ones such as the proposal of another hotel complex were eventually discarded, amid pressure from civil society ( Boissevain, 2004; Briguglio, 2016; TVM, 2020; Visanich, 2022). Residents have also expressed concern about a public contest for the regeneration of the locality arguing that commercial interests were being given priority over the needs of residents ( The Malta Independent (2)).

Residents have also expressed dissatisfaction with the permitted increase in building heights but expressed satisfaction with the number of restaurants and entertainment outlets ( Curmi, 2017). In the meantime, residential, commercial, and touristic development kept taking place in the locality. In Baldacchino’s words,

“The long-term challenge, for Marsascala as much as for Malta as a whole, is to avoid the ‘Benidorm-isation’ of the coastline, with hardly any control or regard for local culture, environment and landscape.” ( Baldacchino, 2012 p.205).

### Methods

As stated in the introduction, the aim of this study is to analyse how Marsascala, a coastal town in Malta, has been urbanized over the years, with particular attention to the development of the tourism industry. For this purpose, a number of research methods were used.

The first was an interpretative qualitative research method which focused on the perspectives of local political elites on the issue under investigation. Five in-depth interviews were held with stakeholders, including residents’ civil society representatives and elected local government officials (from the main governing and the opposition parties in Malta’s two-party system) to collect relevant data. Interviewees were all male and their age varied between 40 and 70 years. Expert sampling, which involves the selection of ‘typical’ and ‘representative’ individuals, was used to recruit interviewees ( Finn *et al.,* 2000). This was possible due to the authors’ respective social networks, which are made further accessible in a hyper personalized small-island state ( Corbett & Veenendaal, 2018). The research method was granted ethical approval by the University of Malta. Interviews lasted over 1 hour and were held online between July and August 2023. Online interviews have been widely used in the field of sociology. This approach is cost and time -effective and ensured that individuals with busy schedules engage in the study causing the least inconvenience ( Thunberg & Arnell, 2022). The use of online interviews has been used in tourism research ( Power *et al.*, 2017) since the use of virtual platforms also permits valid and high-quality interviews ( Suryani, 2013).

No formal questions were prepared; but a checklist of topics derived from the literature review and the research plan was kept in hand to guide the researchers throughout the interview. The major issues tackled included (1) the local economy in the locality, (2) physical changes observed in the built environment over time, (3) how (if any) has the quality of life of residents been impacted and what are the drivers of change, (4) major challenges in the locality, (5) expected impact of development projects planned, and (6) tourism models (if any) acceptable for the local community.

Other secondary data was obtained from major news portals and reports published by authorities and NGOs concerning the area of study. Other portals such as TripAdvisor and Airbnb were used to collect useful information on whether Marsascala still attracts tourism and major attractions.

**Figure 2.**
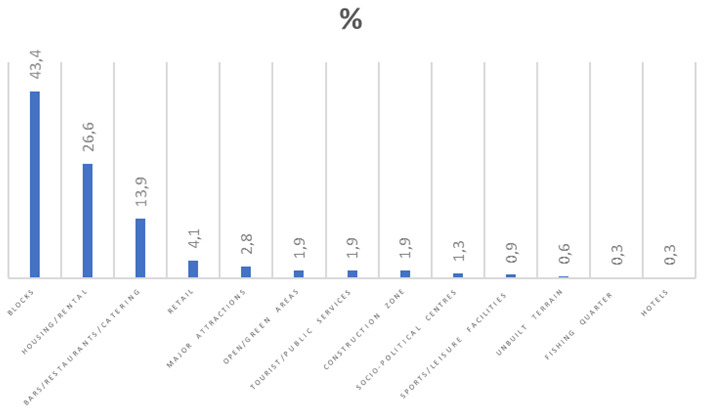
Bar graph showing land use along Marsascala bay.

Another research method made use of visual tools, in line with the recommendations of Sagoe-Addy & Appeaning Addo (2013), and Hernández-Cordero *et al.* (2017). Here orthophotomaps at 20-year intervals (1957; 1978; 1998; 2018) were obtained from the Geomatics Unit, Planning Authority (Malta) and used to observe changes in the development of the locality (See Figure 2). The 1957 image corresponds to the period before the touristic development while the three other photos show various levels of tourism development and degrees of urbanisation.

In addition, a walk-along field trip was carried out during January 2024, where the authors made observations on the use of buildings along the coast of Marsascala Bay. These were categorised into houses, apartment blocks, hotels, bars/restaurants/catering outlets, public/tourist services, major attractions, sports/leisure facilities, unbuilt terrain, open spaces/green areas, retail, and others (including construction sites and socio-political centres). This primary source of data was used to support statements made by interviewees. Some considerations were taken. Buildings with mixed use (example a restaurant at ground floor and apartments on the other floors) were considered separately. During the field trip other observations such as the number of visible tower cranes, activities taking places along the coast and people working or frequenting the area were noted as per Veal (2006).

Data was analysed within the framework of reflexive thematic analysis ( Braun & Clarke, 2022), wherein themes were constructed and engaged upon by the authors in relation to the data in hand.

Limitations of this research method include the possibility of leaving out various perspectives from within the locality, particularly in view of the convenience sample within the elite interviewing framework. This could be mitigated by an accompanying study featuring a representative sample. Another limitation relates to whether social scientific research is taking a broad enough view when studying such matters ( Dahlet *et al.*, 2023). This could possibly be acted upon by “Strengthening the integration of natural and social science research and the effective fusion of the results of that research to inform interested and affected parties of options available to solve common problems.” ( Burbridge, 2020, p.139)

## Results

The results of this study can be grouped into five main themes (see Table 4). These are presented in the following section and are followed by a series of recommendations put forward by the local stakeholders, and subsequently engaged upon by the authors of this article.

**Table 4.** Major themes resulting from the study.

Themes
Tourism plays a major role in the local economy Population increase and overdevelopment are major challenges in the locality More services are available but the quality of life is deteriorating Redevelopment of Jerma Hotel is a major concern A different form of tourism can be offered in Marsascala

Source: Malta Tourism Authority (2)

### Tourism plays a major role in the local economy

The now defunct Jerma Palace Hotel played a central role in the rhetoric of all interviews held. Those interviewed explained that in the past, the Jerma Palace Hotel used to be fully booked all year round and the overspill used to help smaller hotels. Furthermore, several tourists would book half-board and hence go out to eat at one of the local restaurants. When the Jerma closed, small hotels collapsed and restaurants were also badly impacted. However, the locality continued to be frequented by second home owners who are also good tourists for the locality.

In the past 5 years new boutique hotels have opened and old hotels have made refurbishments attracting tourism to the locality. Furthermore, several owners rent extra rooms for short lets through sharing economy platforms (example Booking.com and Airbnb). In fact, a search on Airbnb (November 2023) resulted in over 170 apartments/rooms available for rent. Furthermore, several guesthouses, and apartment blocks used for short-term rentals were observed during the field trip. This is in line with the data provided by Malta Tourism Authority in Table 3 which shows that there are 287 registered beds. One should also keep in mind that the number of beds might be actually higher due to rental apartments which are not declared with national authorities. This shows that the locality still attracts hundreds of tourists mostly in private accommodation and contributing directly to the local community. According to interviewees, the revitalisation of tourism boosted the catering sector.

Other sectors which are gaining ground include water sports. This is confirmed by reviews on TripAdvisor. Other activities and attractions outlined on the portal include diving, Spas, the salt pans, towers, and parks. A heritage trail observed during the field trip listed 17 attractions including chapels and military buildings such as batteries and redoubts. These serve as a pull factor for tourists to frequent the locality.

### Population increase and overdevelopment are major challenges

Interviewees explained how the locality used to have a small population and along with few second home owners from nearby villages such as Zejtun and Zabbar, the locality used to be visited by few tourists, mostly British and ex-Royal Air Force, in the summer period.

However, over the past two decades, the locality witnessed a sharp increase in the population. In fact, the size of the local council - which depends on the size of the population in the respective locality - increased from five members (at its inception in 1993) to 11 members. A member of the local council said that when he first joined the local council, the population was of approximate 7,000 people but has seen the population going up to 17,000. In summer, the population is even bigger as there are still some summer residences, and several rent an apartment to stay close to the sea over the summer period. The Ramla ta’ San Tumas, has become a small village of 2,500 people. A resident explained that until two decades ago, the population of the locality would multiply during the summer months but now this trend has changed and thousands of people are living in the locality all year round.

The increase in population brought with it extensive development. Figure 2 and Table 5 show how land is used along the promenade of Marsascala Bay, as per results obtained through a field trip carried out for this study. During the field trip alone, 15 tower cranes were visible from various points along the bay. A resident said, “This is not development but overdevelopment.” Another interviewee said that there are always excavations taking place and when one tower crane is dismantled, two others are erected.

**Table 5.** Classification of buildings along the coast of Marsascala.

Year	Number of buildings	Percentage %
Houses Apartment blocks Hotels Bars/restaurants/catering outlets Tourist/public services Major attractions Sports/leisure facilities Unbuilt terrain Open spaces/green areas Retail Construction zone Socio-political centres Fishing quarter **Total**	84 137 1 44 6 9 3 2 6 13 6 4 1 316	26.6 43.4 0.3 13.9 1.9 2.8 0.9 0.6 1.9 4.1 1.9 1.3 0.3 100

Source: Malta Tourism Authority (2)

A resident said that Marsacala has a history of being targeted for key projects. One example is the project proposed at Munxar which was abandoned because of objections ( Boissevain, 2004). He added that even the area of Nwadar Park was in the past threatened by the development of the ‘American University of Malta.’ A member of the local council said that the promenade is also under threat. However, the local council objects to structures being built on the promenade. The locality has recently also been earmarked for the development of a yacht marina. A member of the local council said that this would have been a positive step as it would have led to organization of boats and a designated swimmer zone but the way it was presented led to several fears among residents.

While the population had increased, some felt that investment in infrastructure did not match the new demands. There is still the same network of roads and facilities. Allowing additional floors to be built was causing even more challenges. One interviewee said that paying a penalty fee to the Planning Authority for not fulfilling planning policies was not solving any issues with development and lack of parking.

An interviewee said that the rise in population also brought with it challenges with waste management, but this can’t be blamed on foreigners and tourists. While it is not difficult to manage a huge locality with different cultures, building administrators need to do more to inform tenants of waste collection rules. It is worth pointing out that during the field trip no outstanding waste issues were noted along the promenade, however other areas of the locality with a concentration of apartment blocks were less clean and tidy.

Furthermore, the population is now made of various foreigners (see Table 2). This was also observed during the field trip with several foreigners, including third country nationals, both working in the catering sector as well as frequenting other local services. The large Filipino community in Malta even celebrated the feast of Santo Nino in Marsascala ( Times of Malta (5)). Echoing some popular sentiments in the country, some concern was expressed on the increase of foreigners by respondents. According to a resident there is no more a sense of community. Another resident said that overpopulation was a major societal change and criticized politicians for their decisions. “Population is constantly getting bigger - politicians are heading us in the wrong direction.”

### More services are available but the quality of life is deteriorating

As a result of the increase in population, the locality now offers more services including several supermarkets, pharmacies, doctors, and dentists. There is also a new police station and a new school which has some 1,400 students, some 47 different languages and six classes for every year. However a resident spoke against the development of a school and a police station saying these took up few of the remaining open spaces in the locality. He said that both national and local governments were giving a bad example. Supporting this argument, another interviewee said that open spaces and green areas have shrunk and criticized authorities for chopping mature trees to make space for other infrastructure. Similarly, a small fuel station made an extension taking up further green space.

A member of the local council said “Luckily we have the promenade where to walk but even this is under threat from kiosks which to date have been successfully blocked by the local council.” While services in general increased, they are not enough for the population size. For example, the public bus service is not sufficient for demand in certain hours.

In addition, the locality was never planned to have seven stories of buildings in certain areas and such a huge population. As a result, there is a big issue with parking. A resident said that the rise in population and traffic had also caused the traditional vegetable vendors to be removed from the square to facilitate traffic management, altering the character of the village.

According to residents the quiet environment related to the locality had vanished as excavation works were constantly taking place. The constant presence of tower cranes has also changed the landscape of the locality. In this regard, one resident said “I want to live in peace, I want to enjoy my property. I do not care that my property has more value.”

A common sentiment expressed by respondents was also about the few open/green spaces, and how this has negatively impacted their quality of life. Indeed, high quality public open spaces have been found to contribute positively to people's quality of life ( Beck, 2009; Nasution & Zahrah, 2014).

### Redevelopment of Jerma Hotel is a major concern

Overdevelopment and lack of planning is a major concern for locals. However, the redevelopment of the Jerma Hotel is even more controversial. An interviewee said that the redevelopment of Jerma Hotel is a complex issue. In the past, it was needed for the Southern part of Malta as it used to serve professionals who visited the dockyard or close by industrial estates. Furthermore, according to a member of the local council the Jerma Hotel used to attract a niche of tourists who used to look for a quiet place and who travelled to Malta to swim, eat and relax. It was also a place where local communities could meet. However, its new proposed development follows recent trends that merge real estate with the development of the hotel to make the project feasible.

One resident said “The problem in Marsascala is not an issue of bad planning but an issue of no planning.” The area known as Siberia (where the Jerma hotel will be redeveloped) has developed and now has over 10,000 residents but there is no green /open space. As a result, some locals do not want to have a hotel and prefer if the government takes back the land to convert it into an open space for the local community. Another interviewee supported the project saying that plans include a central square open for the public and this will add an open space for the residents.

A big fascia wants the hotel to be rebuilt without any real estate if public space is to be sacrificed. According to a study conducted in 2014, most locals, businesses and authorities regard the overall tourism impact as positive and agree with the decision of placing Marsascala back on the tourist map. Furthermore, they also expressed their wish to see the revitalisation of the former four-star hotel and/or building of other hotels to regenerate tourism in the locality ( Hopkins, 2014). One resident said that land was originally given to the private sector for tourism purposes and not real estate, but the new project will include real estate, not just a hotel.

Others have a different view. One interviewee said “We ruined almost all Malta, now we want to ruin here too?” He said that several believe that the aim is to build eight floors and later make a request to build additional floors causing further problems in the locality. Another interviewee said that while it may be positive for the economy, it will cause further traffic and increase the parking problem. “The bypass is already jammed and the hotel will bring further traffic to the locality.”

### A different form of tourism for Marsascala

In general, the respondents are not against tourism. In fact, some interviewees expressed disappointment that the hop on hop off bus does not include Marsascala in its itinerary to bring tourists to visit the locality.

There is agreement that the locality can offer a different form of tourism than other parts of the island. The locality is still home to some fishermen and attracts tourists because it is different. One interviewee said “This is why we did not want the marina, not to be similar to other localities. Let's use this different character to attract tourists.” 

Some want to focus more on sea-based tourism. The locality already has a diving centre and wrecks off the bay which are an attraction for diving enthusiasts. Other suggestions have been to restore the St Thomas tower and repurpose it as a tourism attraction and to value the saltpans found along the coast of the locality. As outlined on TripAdvisor, certain attractions can only be observed from the outside.

The locality also boasts of the Magħluq Natura 2000 site and the Nwadar national park where afforestation of some 1000 trees is being planned. A resident said that the park needs to be well maintained as some trees were planted but died later on. “Nwadar can become a small Mizieb” In addition Marsascala is home to the family park. The site is an interesting place as no further waste management is taking place in the adjacent plant.

***Recommendations***

Interviewees made several recommendations to address current issues. One resident suggested the implementation of a 5-year moratorium on building to ease overpopulation, overdevelopment, and traffic. ‘Freezing development’ has also been proposed by Young (1983) to prevent the village from losing its characteristics.

Another suggestion was to invest further in infrastructure to cater for the rise in population. A member of the local council said that the rise in population merits further investment and upgrade in existing infrastructure, not least the promenade. Further investment along the front can lead to the development of a walk from Nwadar Park to Munxar. Such investment will alleviate the issue of lack of open spaces, even if existing ones were not considered by respondents to be enough to balance the ongoing urban sprawl. Policymakers need to take this into account when deciding the fate of proposed mega projects and use of open spaces.

Local councillors also suggested the need to support boutique hotels instead of the big hotels describing the former as more ideal since they cause less impact on the locality. Short lets are also considered positive for the locality as tourists are distributed throughout the locality and not concentrated in one area.

Such recommendations are largely in sync with the list of recommendations of the Marsascala Residents’ Network ( Times of Malta (2)). In particular, the network is suggesting that “the seaside should be viewed as a natural home and a host for residents and visitors respectively, where sustainable tourism can develop environmentally”; “The provision of high-quality, affordable mixed housing”; smart interventions for traffic “without causing detriment to the locality’s natural environment”; “A comprehensive methodology to assess the rising sea levels and severe flooding problems needs… to identify effective preventative investments”, and “Finally, creativity, sustainability and well-being must be put at the heart of the proposals, as a means of diversifying and growing the local economy and as an accessible way of getting the whole community on board.”

### Orthophoto maps confirm transformation of locality

Orthophoto maps obtained confirm the complete transformation of the locality as outlined by the interviewees. The image from 1957 ( Figure 3a) corresponds to pre-tourism development and can be considered as what Young (1983) refers to as the ‘Late Traditional’ stage. The figure from 1978 ( Figure 3b) corresponds to ‘Early Tourism Involvement’ whilst the figure from 1998 ( Figure 3c) can be attributed to the phase ‘Expanding Tourism Development’. The most recent orthophoto available from 2018 can be linked to the ‘Intensive Tourism-Consolidation’ phase ( Figure 3d). Incidentally, Young’s model had foreseen the development of a marina at this stage, a project which was indeed proposed in 2021 and abandoned following campaigning from NGOs and civil society ( Times of Malta (4)). Comparing the 1957 map (pre-tourism development) and the 2018 map one can observe a complete transformation of the locality with extensive urbanisation that has altered the coastal town in line with what interviewees have outlined.

**Figure 3a.**
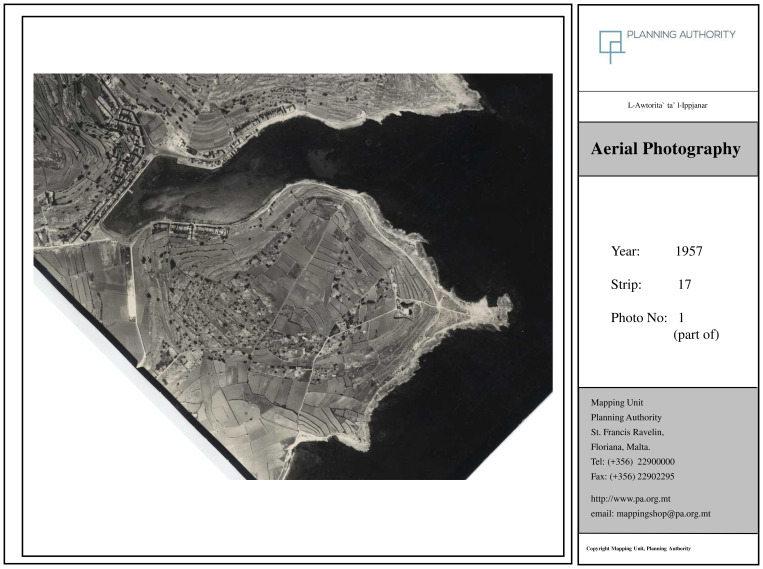
Map showing the locality of Marsascala in 1957. Photo provided by the Geomatics Unit, Planning Authority.

**Figure 3b.**
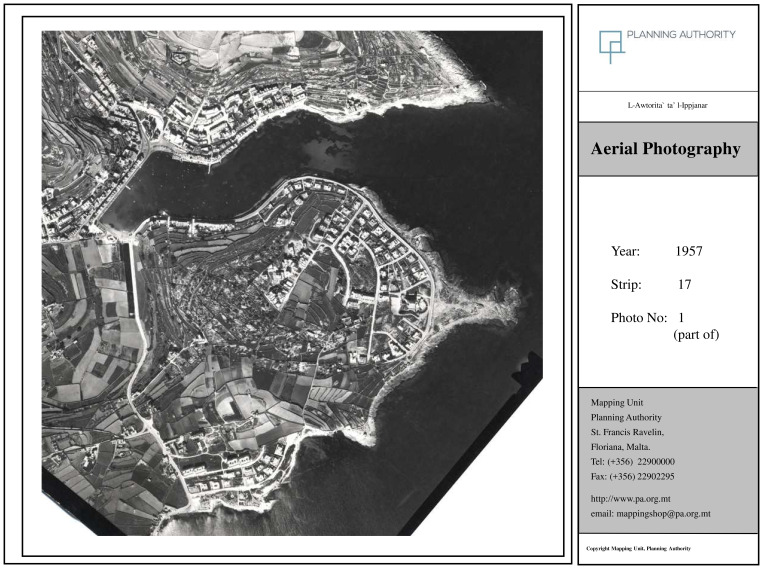
Map showing the locality of Marsascala in 1978. Photo provided by the Geomatics Unit, Planning Authority.

**Figure 3c.**
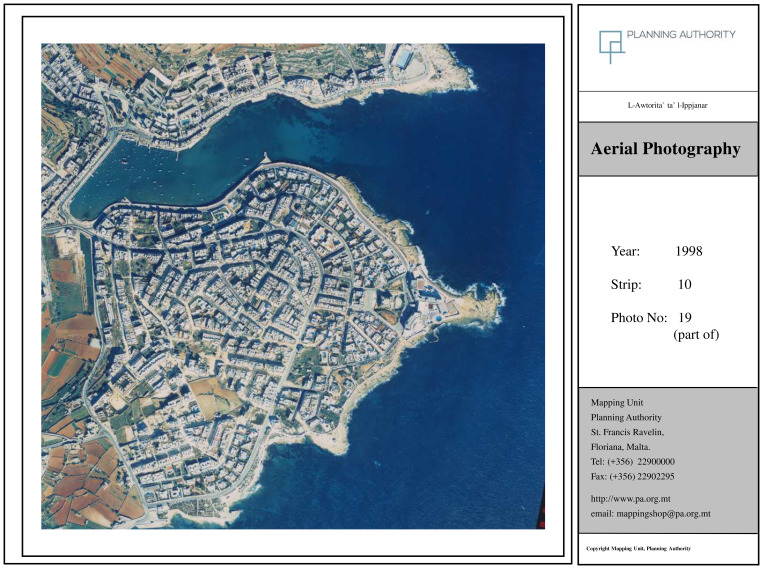
Map showing the locality of Marsascala in 1998. Photo provided by the Geomatics Unit, Planning Authority.

**Figure 3d.**
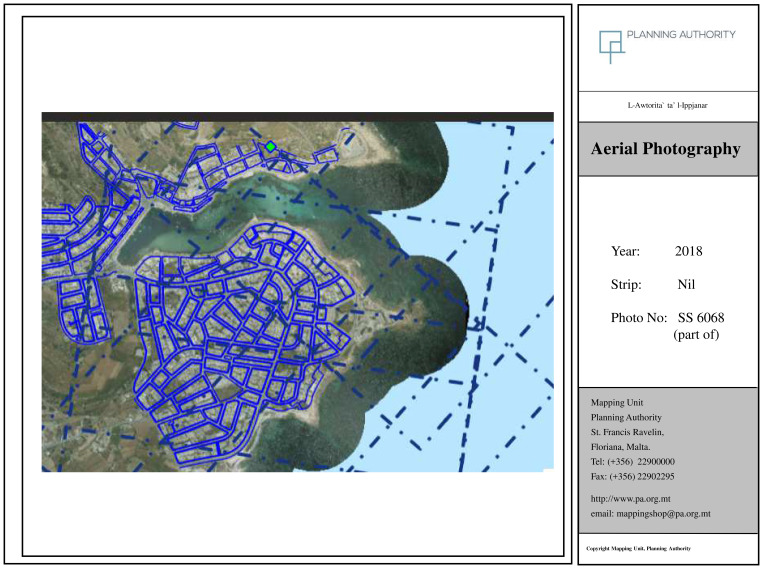
Map showing the locality of Marsascala in 2018. Photo provided by the Geomatics Unit, Planning Authority.

## Discussion

Tourism development in Marsascala, demand for short lets as well as accommodation for foreigners (including those working in the tourism and hospitality sector) triggered development of (almost) the entire coastal area and further inland of the locality. This development not only contributed to urbanisation but also brought with it a number of challenges including over population, traffic, lack of green spaces and deterioration of quality of life. Similarly, Crespi-Vallbona and López-Villanueva (2024) argue that damages resulting from touristification include “an increase in the number of tourist apartments to the detriment of housing for residential use; an increase in the purchase and rental prices of these properties; loss of local product establishments and conflicts in the coexistence between the spaces.

Referring to his model, Young (1983) argues that if the tourist product reaches stage 6 (intensive tourism consolidation) tourism would be seriously weakened or diminished. Young had also raised a series of questions, in particular what impact this will have on the quality of life. Stakeholders interviewed indicate not only that this stage of tourism development and urbanisation has been reached but also that the quality of life has deteriorated considerably.

Furthermore, Young had also asked what stage would follow ‘intensive tourism consolidation.’ We are hereby proposing a 7th stage which can be analysed further in subsequent studies: ‘real estateisation’. Here, we are proposing that more areas which previously served for other purposes start to merge or gradually be taken over by real estate projects, as is the case with the proposed redevelopment of the Jerma Hotel into a tourism and real estate project ( Malta Today (1)).

Various residences are already used to host non-Maltese nationals, whether as tourists or residents, in what looks like a hybrid process of real-estate, urban, and touristic development. Gil (2023), refrains from calling this touristification or gentrification and instead refers to this model as short-term rental housing assetization strategy. This leads to speculation and increase in property prices as short-term rentals induce rent gaps. This explains why property prices keep increasing in Malta, not least in the Southern region ( Times of Malta (6)). In this regard, and with respect to literature on gentrification, we propose that the processes of development characterising Marsascala are more in line with Gil’s analysis, particularly in a society where, according to the National Census ( National Statistics Office – Malta (4)) almost 75 % of Maltese individuals own their main residence, whilst almost 53% per cent of non-Maltese residents rented furnished accommodation, and where the majority of housing stocks were flats or penthouses (in Marsascala, these amount to 4,614, in comparison with 571 Terraced houses, 210 Semi/Fully-detached houses, 1592, Maisonettes, and 76 Others ( National Statistics Office – Malta (4)) At the same time, it must be emphasized that the price of property is becoming increasingly unaffordable for prospective buyers/tenants, and high rents have been cited as the major reason for Malta’s high turnover rate of foreign workers ( Malta Today (2); Malta Today (3)).

This phenomenon requires further quantitative and qualitative social-scientific evidence on the usage of such properties. We are aware of at least one study currently being carried out in Malta in this regard. It is also interesting to note that Malta’s reliance on foreign residents is also exemplified by the fact that the country’s passport is ranked joint fifth most powerful in the world ( Times of Malta (7)), though many foreign workers, especially in lower paying jobs, do not afford such luxury. 

The touristization and real estateisation process of Marsascala can be contextualised within the challenges of the Maltese tourist industry and beyond. In this regard, various recommendations have been made by a group of experts at the University of Malta, “in the quest for sustainable tourism which is defined by the UN Environment Programme and UN World Tourism Organization as “tourism that takes full account of its current and future economic, social and environmental impacts, addressing the needs of visitors, the industry, the environment and the host communities.” ( Avellino *et al.*, 2023). The authors of this article are not aware of any sustained and holistic attempt by Maltese governments to follow recommendations of the sort.

Here one could also refer to the importance of tools such as social impact assessments, which can be defined as “includes the processes of analysing, monitoring and managing the intended and unintended social consequences, both positive and negative, of planned interventions (policies, programs, plans, projects) and any social change processes invoked by those interventions. Its primary purpose is to bring about a more sustainable and equitable biophysical and human environment.” ( International Association for Impact Assessment - IAIA). In this regard, some ad hoc social impact assessments and surveys were carried out on development proposals, including two controversial ones at Marsascala which were eventually withdrawn (the American University of Malta and the Yacht Marina respectively), and one can also refer to the 2019 policy development wherein the competencies of Regional Councils include the social aspect, which, in turn, includes researches and report of social impact evaluations. ( European Committee of the Regions, n.d.). At the same time, Social Impact Assessments, particularly as recommended by the IAIA, are as yet not mainstreamed in Maltese policy making.

Such tools can help us understand the interactions such as those between the built environment and the well-being of local communities. Various strategies have been proposed to improve the well-being of local communities including improving public transport while restricting cars, include forms of urban nature and public spaces, maintain the upkeep of public areas and vegetation, as well as develop aesthetically pleasing buildings and public spaces based on residents' needs and preferences ( Mouratidis, 2021).

From a more critical perspective, one could also situate the social changes under analysis within a treadmill of production ( Gould *et al.*, 2008) which is dependent on consistent economic growth to feed the needs of the system and its dominant social classes. In this regard, a symbiotic relationship between developers and the State, could be theorized. Here developers provide economic growth and other incentives. The State provides policy and operational support. This relationship rests on the ideological commitment to such development through the exploitation of land ( Briguglio, 1998). Here, we note that current development processes of the Marsascala type are even more nuanced in view of multi-directional impacts, such as, on the one hand, the capitalization of assets even by small-scale owners, and on the other hand, the spread of housing precariousness in view of unaffordable prices amongst both Maltese and foreigners.

Both these reformist and critical perspectives can help us locate the study within Sustainable Development Goals. Indeed, this paper contributes to the following sustainable development goals respectively, 3 (Good Health and Wellbeing); 8 (Decent Work and Economic Growth); 11 (Sustainable Cities and Communities); and, to a lesser extent, 14 (Life Below Water); and 15 (Life on Land), in particular with respect to quality of life, urbanization and respective social, environmental, and economic impacts.

## Conclusion

By assessing the impacts of coastal tourism and related activites, this paper provides an empirical case study that falls within the purpose of the RethinkBlue Cost Action.. In this regard, th locality of Marsascala has experienced extensive urbanisation over the years. This has been initially triggered by second homes and tourism. In recent years, the locality has continued to experience further urbanisation to cater for an increase in residents who live in the locality all year round, due to short-term rentals as well due to the demand for accommodation by foreigners who work in various sectors including the tourism and hospitality sector. This brought with it benefits including additional services but also a series of challenges for residents including traffic, lack of parking, lack of green/open spaces and inconvenience due to construction projects which in return led to deterioration of the quality of residents.

The locality has not only reached stage 6 (intensive tourism consolidation) of Young’s model ( 1983) but risks to pursue the route to what the authors refer to stage 7 ‘real estateisation’ whereby tourism facilities and other areas in the locality are taken over by real estate projects fuelled by national demand for housing. Considering that several houses/second homes are still present along the coast, this trend may lead to further agony to the local community as such dwellings make space for further blocks. As Gil (2023) outlines, as long as tourist demand continues to grow and regulations allow for the development of short-term rentals, investors and landlords will convert large numbers of properties and switch capital to this model. While the local community together with civil society has shown resilience to constant threats, there is still the risk that what remains of the character of the village will be lost to the detriment of touristic appeal. This needs to be taken into account by policy makers especially in terms of urban planning and tourism development as the island seeks to go beyond the 3 million annual visitors.

## Ethics and consent

Research was conducted after submitting a Research Ethics Review Procedures (REDP) Form ARTS-2023-00296 to the Faculty Research Ethics Committee (FREC) Arts which reviewed the application and determined that the research is in conformity with the University of Malta’s Research Code of Practice. Informed consent was obtained from all subjects involved in the study. The interviews were held online, and the invitation was sent to the participants via email or WhatsApp, together with a disclaimer form as well as a recruitment letter, providing information on the research and how data collected will be used. Given that the interviews were held online, it was more practical to obtain verbal consent. Before each interview, participants were asked to grant consent as per document circulated reminding them that they are free to accept, refuse or stop participation at any time without giving any reason. Once participants confirmed their willingness to participate in the study, the researcher started to field the questions.

## Data Availability

Data collected through interviews is available in the results section. As outlined in the methods section, interviews were held online and notes were taken. These notes were fully integrated in the results section removing duplicated content and merging points raised in the process. The draft notes were later erased to ensure confidentiality. Therefore, there is no additional data to be shared.
