# Supplementary material for: Tourism, Real Estate, and Urban Pressures: The case of Marsascala, Malta
Source: Open Res Eur. 2025 Aug 29;4:183. Originally published 2024 Aug 15. [Version 2] doi: 10.12688/openreseurope.17641.2 (PMC12413615; doi:10.12688/openreseurope.17641.2)
Supplement: Peer review history

# Reviewer report: Reviewer response for version 2

**DOI:** 10.21956/openreseurope.22782.r59288

**Published:** 2025-09-10

**Authors:** José Antonio Barragán Ojeda

**Affiliations:** 1 ENES-Mérida, UNAM, Merida, Mexico

I agree with the current version of the article. The authors made the relevant changes, and I believe the text meets the requirements of the previous review.

If applicable, is the statistical analysis and its interpretation appropriate?

Partly

Is the study design appropriate and is the work technically sound?

Yes

Is the work clearly and accurately presented and does it engage with the current literature?

Partly

Are the conclusions drawn adequately supported by the results?

Yes

Are sufficient details of methods and analysis provided to allow replication by others?

Yes

Are all the source data and materials underlying the results available?

Yes

Reviewer Expertise:

Human geography, Tourism Geography, ecotourism

I confirm that I have read this submission and believe that I have an appropriate level of expertise to confirm that it is of an acceptable scientific standard.

# Reviewer report: Reviewer response for version 2

**DOI:** 10.21956/openreseurope.22782.r59287

**Published:** 2025-09-05

**Authors:** José Alberto Rio Fernandes, Jorge Ricardo Pinto

**Affiliations:** 1 University of Porto, Porto, Portugal

The article has been significantly improved.

 I keep my criticism on the methodological approach, especially on the fact that several conclusions are based on just 5 interviews. With a note on that limitation in the text, I think that it may be indexed.

If applicable, is the statistical analysis and its interpretation appropriate?

Yes

Is the study design appropriate and is the work technically sound?

Partly

Is the work clearly and accurately presented and does it engage with the current literature?

No

Are the conclusions drawn adequately supported by the results?

Partly

Are sufficient details of methods and analysis provided to allow replication by others?

Yes

Are all the source data and materials underlying the results available?

Yes

Reviewer Expertise:

Urban geography. Urbanism.

We confirm that we have read this submission and believe that we have an appropriate level of expertise to confirm that it is of an acceptable scientific standard.

# Reviewer report: Reviewer response for version 1

**DOI:** 10.21956/openreseurope.19061.r43419

**Published:** 2024-10-21

**Authors:** José Antonio Barragán Ojeda

**Affiliations:** 1 ENES-Mérida, UNAM, Merida, Mexico

the topic of study is pertinent and appropriate, but part of the literature used is obsolete. An update of the Young (1983) model is understood and appreciated, but it is necessary to compare other models such as Butler (2008), Knofou (1999), Ojeda (2024), Murray and Blazquez (2011) among others.

 Blázquez, M., & Murray, I. (2011). [Ref 5]

 Ojeda, A. B. (2024).[Ref 1]

 Butler, R. (1980). [Ref 2]

 Butler, R. (2008). [Ref 3]

 Knafou, R. (2017). [Ref 4]

 I declare that the article I reviewed raises a very well-founded issue. However, I consider that the research development was insufficient, so I propose to accept it with serious modifications.

If applicable, is the statistical analysis and its interpretation appropriate?

Partly

Is the study design appropriate and is the work technically sound?

Yes

Is the work clearly and accurately presented and does it engage with the current literature?

Partly

Are the conclusions drawn adequately supported by the results?

Yes

Are sufficient details of methods and analysis provided to allow replication by others?

Yes

Are all the source data and materials underlying the results available?

Yes

Reviewer Expertise:

Human geography, Tourism Geography, ecotourism

I confirm that I have read this submission and believe that I have an appropriate level of expertise to confirm that it is of an acceptable scientific standard, however I have significant reservations, as outlined above.

## References

[1] : Análisis geohistórico de la turistificación de Quintana Roo en México, en los últimos cien años (1920-2020). *Revista de geografía Norte Grande* .2024; 10.4067/S0718-34022024000100105 1-22 10.4067/S0718-34022024000100105

[2] : THE CONCEPT OF A TOURIST AREA CYCLE OF EVOLUTION: IMPLICATIONS FOR MANAGEMENT OF RESOURCES. *Canadian Geographies / Géographies canadiennes* .1980;24(1) : 10.1111/j.1541-0064.1980.tb00970.x 5-12 10.1111/j.1541-0064.1980.tb00970.x

[3] : The Tourism Area Life Cycle in the Twenty‐First Century.2004; 10.1002/9780470752272.ch13 159-170 10.1002/9780470752272.ch13

[4] : Le tourisme réflexif, un nouveau fondement d’un tourisme durable. *Arbor* .2017;193(785) : 10.3989/arbor.2017.785n3001 10.3989/arbor.2017.785n3001

[5] : A geohistory of the touristification of the Balearic Islands. *https://www.redalyc.org/articulo.oa?id* .2010;

# Reviewer report: Reviewer response for version 1

**DOI:** 10.21956/openreseurope.19061.r43418

**Published:** 2024-09-19

**Authors:** Melanie Kay Smith

**Affiliations:** 1 Budapest Business University, Budapest, Hungary

This is a really interesting study and the authors make a good effort to provide a critical analysis of tourism development in the location. However, the central narrative needs to be explained a bit more clearly and carried through to the end. It seems that the article is mainly about 'no planning' and the consequences of that, but it would be important to clarify this from the start. Then it would be logical to explain why gentrification and touristification take place, including the growth of unsuitable forms of accommodation provision (or at least, those that can become problematic like Airbnb). The lack of clear narrative/focus means that the Abstract is a bit disjointed. Unfortunately, many of the issues relating to overtourism, gentrification, STRs, etc. have become so widespread and complex that it is hard to do justice to them in a couple of paragraphs. The result is that the Literature Review seems rather superficial and random. This is why a more focused narrative is needed (e.g. the shortcomings of top-down planning or no planning). Please unpack and discuss this sentence, which could hold the key to the narrative: "urban development, gentrification, and mobility interact with planning and the two are mutually constitutive".

 I would welcome a brief typology of coastal destinations and the differences between them. Please contextualise Malta within this typology. After all, a Mediterranean coastal resort/island is very different from a Baltic or North Sea destination, and it is questionable whether Asian coastal destinations are directly comparable (are they?). For example, is the "new socio-economic climate" developing everywhere? If so, please explain how/why (e.g. the impact of SDGs or other international factors?).

 The Young model works well in this context, although many authors select Butler and variations of the lifecycle model instead. The authors might want to explain in slightly more depth why they chose Young's model instead.

 The selected research methods are quite novel, especially the use of photomaps. Slightly more analysis of these would be welcome.

 The interviews are a bit limited and offer mainly a 'top-down' perspective. The authors should somehow relate this choice of method to the central narrative about planning (which tends to be 'top-down' in many cases).

 Overall, the explanation of research methods is rather rushed and it would be useful to explain in more detail and more systematically how portals like TripAdvisor and Airbnb were analysed. The information yielded is nevertheless interesting. Reviewer comments could also have provided some insights, although the article is rather about planning than tourists' experiences.

 The references to SDGs is a bit tokenistic at the end and should be more integrated into the findings and earlier discussions. 

 Figure 2 is not very readable.

If applicable, is the statistical analysis and its interpretation appropriate?

Not applicable

Is the study design appropriate and is the work technically sound?

Partly

Is the work clearly and accurately presented and does it engage with the current literature?

Partly

Are the conclusions drawn adequately supported by the results?

Yes

Are sufficient details of methods and analysis provided to allow replication by others?

Partly

Are all the source data and materials underlying the results available?

No source data required

Reviewer Expertise:

cultural tourism, urban planning, city tourism, health tourism, wellbeing

I confirm that I have read this submission and believe that I have an appropriate level of expertise to confirm that it is of an acceptable scientific standard, however I have significant reservations, as outlined above.

# Reviewer report: Reviewer response for version 1

**DOI:** 10.21956/openreseurope.19061.r43420

**Published:** 2024-09-10

**Authors:** José Alberto Rio Fernandes, Jorge Ricardo Pinto

**Affiliations:** 1 University of Porto, Porto, Portugal

I read with interest the article.

 To associate tourism with urban development in the XXI century was a promising idea.

 However, the authors fail to deliver. I try to explain why. And I begin with the two main problems:

 1. When taking Young (1985) model as central it is mandatory that:

 - the linear evolution of "touristization" and landscape change is debated. It has to be noted the possibility of tourism territories to be rejuvenated and other nonlinear evolution possibilities;

 - the model is outdated. It has to be noted that it dates back to 1985 when there was no "platform economy" (AirBnB, Uber, ...), the globalization process was not so intense, low-cost flight were not what they are today... The world changed a lot in 40 years! Anachronism is a big scientific error. Also, on the linear scientific approach to tourism I don’t understand why the authors fail to mention f Butler cycle of the tourism product.

 2. The theoretical chapter is very fragile and not coherent with the following chapters. 

 - What is overdevelopment? Is there a measure for "adequate development" - there's no discussion about this;

 - What’s overpopulation? Is Bangladesh overpopulated? London? Malta? …

 - What is touristification? Just the hybrid combination of tourism and gentrification? Touristification can’t happen without gentrification, only by increase of low-middle class tourism? There’s no discussion on concepts and processes…

 Only one page and some lines for literature revision is not enough and all the discussion is rather vague and fails to debate what becomes fundamental in the Discussion, globalization, and the increasing relevance of real estate multiscale investment in local areas.

 Also, curiously, in the last lines of the conclusion, the authors express their concern about what is left of the character of the village. However, nowhere in the article the concept of character is discussed, as the idea of its dynamic dimension is completely forgotten.

 3. The text is rather descriptive after the Revision of the Literature and before Discussion. It should be more reflexive.

 4. There are much other aspects that should not appear on a text before being indexed. I am sure the authors can easily improve:

 - punctuation and small mistakes (“, As” in p. 2; “th” in p. 21)

 - avoid the mention to "qualitative interviews", in the absence of quantitative interviews (p. 1);

 - include comparative statistics of others islands as Baleares, Canary, Creta and/or Madeira before (or as) presenting “Tourism in Malta” (pp. 5,6);

 - avoid the expression “l phenomenon of lack of sustainability” (p. 6);

 - the presentation of and use with the inclusion of a map (p. 11);

 - that conclusions from a 10 years old study should not be taken as actual (p. 15);

 - the mention of Nwadar Park and Munxar being illustrated with a map (p. 16);

 - by considering all maps of fig. 3 in the same format and scale.

If applicable, is the statistical analysis and its interpretation appropriate?

Yes

Is the study design appropriate and is the work technically sound?

Partly

Is the work clearly and accurately presented and does it engage with the current literature?

No

Are the conclusions drawn adequately supported by the results?

Partly

Are sufficient details of methods and analysis provided to allow replication by others?

Yes

Are all the source data and materials underlying the results available?

Yes

Reviewer Expertise:

Urban geography. Urbanism.

We confirm that we have read this submission and believe that we have an appropriate level of expertise to state that we do not consider it to be of an acceptable scientific standard, for reasons outlined above.
